# A Complex Surgical Case of a Morel-Lavallee Lesion

**DOI:** 10.7759/cureus.48764

**Published:** 2023-11-13

**Authors:** Dylan W Hefner, Makenzie Dye, Rebekah Lantz

**Affiliations:** 1 School of Medicine, Wright State University Boonshoft School of Medicine, Dayton, USA; 2 Internal Medicine, Miami Valley Hospital, Dayton, USA

**Keywords:** morel-lavallee lesion, unrestrained driver, vehicle ejection, poorly controlled medical conditions, i&d- incision and drainage, thigh bleed, police chase, mva (motor vehicle accident), trauma, internal denudement

## Abstract

A Morel-Lavallee lesion (MLL) is a rare internal denudement injury of skin and hypodermis from deep fascia, usually occurring hours to days after an inciting trauma. A common location is the pelvis or thigh where there is prominent vascularization and may mimic diagnoses such as deep vein thrombosis or contusion. Fluid collections that persist despite conservative management require surgical intervention and frequent and prolonged hospitalizations as in this case of a patient with a persistent MLL. We emphasize early imaging for diagnosis and surgical service involvement, as delay may lead to persistent symptoms and worse health outcomes.

## Introduction

Morel-Lavallee lesions (MLLs) arise as a post-traumatic accumulation of hemolymphatic fluid between superficial and deep fascial planes [1]. Tangential forces applied to superficial soft tissues with greater mobility relative to underlying deep tissue cause tearing of arterioles, venules, and lymphatic vessels spanning the facial plane, leading to a fluid accumulation confined by the fascia [1]. Effusions may develop quickly or slowly, depending on the involvement of arterial beds or lymphatics, respectively [1]. Blood is eventually reabsorbed, replaced by serosanguineous fluid, and the lesion becomes lined with a fibrous capsule, contributing to the chronic nature of the lesion [2]. Commonly involved areas include the thigh or pelvis, but they can also be caused by low-velocity crush injuries and contact sports [3].

The trochanter and upper thigh are the most commonly involved regions where there is a rich vascular plexus supplying blood from the dermis to the fascia lata [2]. Locations of higher vascularity are at greater risk and those of lesser vascularity are at lower risk. Therefore, less common sites include the gluteal and lumbosacral regions, and uncommon sites include the abdominal wall and calf [1]. Age can also depict locations at risk of fluid collection. Adults are more likely to collect seroma, abscess, or hematoma after trauma in the hip and thigh, while the knee and distal lower extremity are more likely in children [4].

MLLs are rarely seen in practice, as they often go undiagnosed or are diagnosed late [5]. Prevalence is associated with 8.3% of acetabular fractures and in a male-to-female ratio of 2:1, where men appear to experience more polytrauma [4]. There are six subtypes based on imaging appearance, chronicity, and tissue composition according to Bonilla-Yoon et al., [1] as shown in Table 1 (Appendix). Differentials should initially include seroma, abscess, and solid soft tissue masses [4]. 

While ultrasound is useful in identifying supra fascial fluid accumulation, computed tomography (CT) will definitively show the presence or absence of fluid [4]. Meanwhile, magnetic resonance imaging (MRI) better characterizes density and extension into the surrounding soft tissue [4]. Most acute and chronic MLLs ≤400 mL resolve with percutaneous aspiration and sclerodesis using doxycycline solution, followed by four weeks of compression bands [6,7]. Lesions >400 mL require open incision and drainage (I&D), with dead space closure and quilting sutures. Low suction drains are also employed in situ till drainage <30mL over a day [7].

We present a case of a 28-year-old male who presented with persistent pain and left leg swelling three weeks status post trauma. The inciting factor was a high-speed chase pursuit with a motor vehicle accident, and the patient was ejected through the front windshield as an unrestrained driver. Along with the rarity of MLLs, an interesting feature of the case includes a complex course despite appropriate imaging and standard-of-care treatment. He had recurrence requiring multiple incisions and drainage.

## Case presentation

A 28-year-old African American man with history significant for obesity, diabetes, and hypertension initially presented in October 2022 after being the pursuant unrestrained driver of a high-speed police chase, wrecking his vehicle and being ejected 50 feet from the car. He was admitted for contusions, strain injury, and traumatic rhabdomyolysis without fracture, and his primary conditions were poorly managed as an outpatient. His abbreviated injury scale as appreciated by trauma service was points for minor injury to head and neck (+1), minor injury to abdomen (+1), moderate injury to left lower extremity (+2), and minor external injury (+1). The injury severity score as calculated by the squares of each equaled 9 out of 75 total possible points, indicating overall minimal risk for complication and mortality. He had no personal or family history of coagulopathies and no labs indicating this effect. He had a normal coagulation panel and hemoglobin/hematocrit levels. Rhabdomyolysis improved with IV fluids and he was treated for cellulitis. At that time, there was clinical suspicion of a left medial meniscal tear due to an equivocal McMurray exam but no surgical intervention was performed due to the inability to confirm diagnosis.

He presented three weeks later with left leg swelling and pain, ambulating with the use of a crutch and knee immobilizer, and had no infectious symptoms. He experienced persistent and worsening swelling in his left thigh with worsening pain over time. He denied pain in the calf and was able to ambulate at this time. Exam was significant for the hard left upper thigh which wrapped around medially to the knee and was associated with tense pressure. There were no external lesions or entry points for infection. Pulses were intact. Venous Doppler ultrasound was obtained to assess for compartment syndrome. Findings were negative for clot however concerning for extensive hematoma from hip to knee.

**Figure 1:**
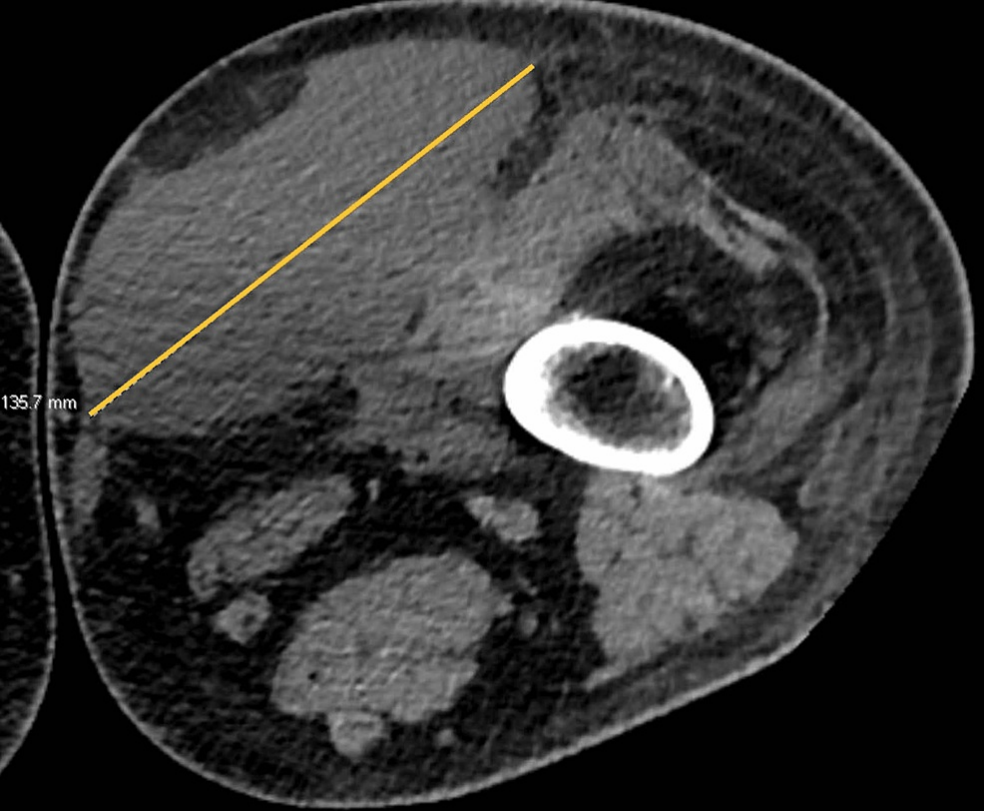
October 2022 CT left lower extremity with contrast shows large anteromedial right thigh deep subcutaneous fluid collection, 43.4 x 13.6 x 5.4 cm in craniocaudal/transverse/anteroposterior dimensions, likely representing the Morel Lavallee lesion. This image shows the transverse dimension.

CT extremity further defined the lesion as Morel-Lavallee (Figure 1). Surgery was consulted and bedside aspiration was performed after prophylactic cefazolin 2 gm IV was administered. Skin over the incision site was prepped with betadine and chlorhexidine. Anesthesia was obtained by infiltration using 2% lidocaine without epinephrine. With the patient in the supine position, an 18-gauge needle was inserted over the medial distal thigh and repeated draining of dark serosanguinous fluid using a 30-cc syringe was performed. The leg was massaged in a fashion that allowed for the fluid to drain distally toward the needle. After 1800mL of fluid was drained, the patient was asked to sit on the edge of the bed and an additional 100mL was drained by gravity. The medial distal thigh was prepped, and another needle stick drained an additional 100mL of dark serosanguinous fluid. No cultures were sent at this time as there was negligible suspicion of infection. The patient was then cleaned with a wet-to-dry method. The two needle puncture sites were covered in a non-adherent dressing and covered in a roll of Kerlix gauze. The patient’s leg was then tightly wrapped in an Ace bandage from the groin to toes. The patient was provided with home bandaging supplies and instructed on how to adequately wrap the leg. Instructions for left leg care were placed in care. Follow-up in one week and continued use of compression wrap from groin to toes were recommended.

**Figure 2:**
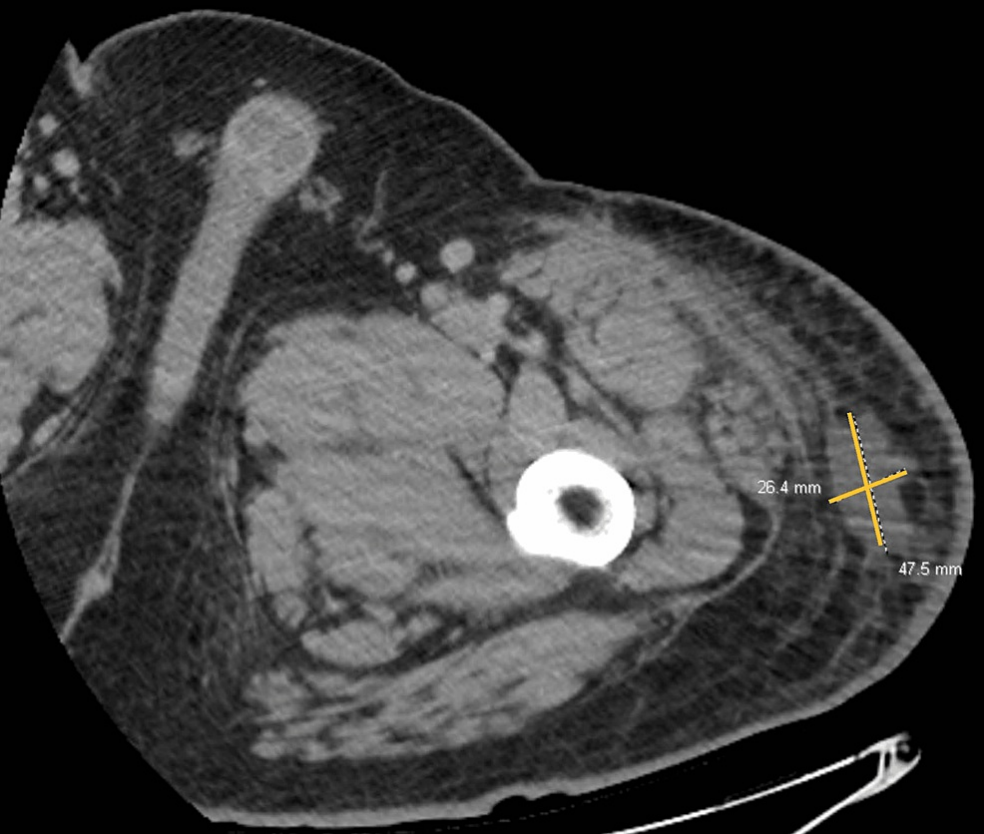
November 2022 CT left lower extremity with contrast status post-irrigation and debridement of left anteromedial thigh hematoma shows residual collection of gas and fluid measuring 4.8 x 2.1 x 40.0 cm, previously 13.6 x 5.4 x 43.4 cm. This image shows residual fluid collection 4.8 x 2.1 cm in the transverse dimension.

**Figure 3:**
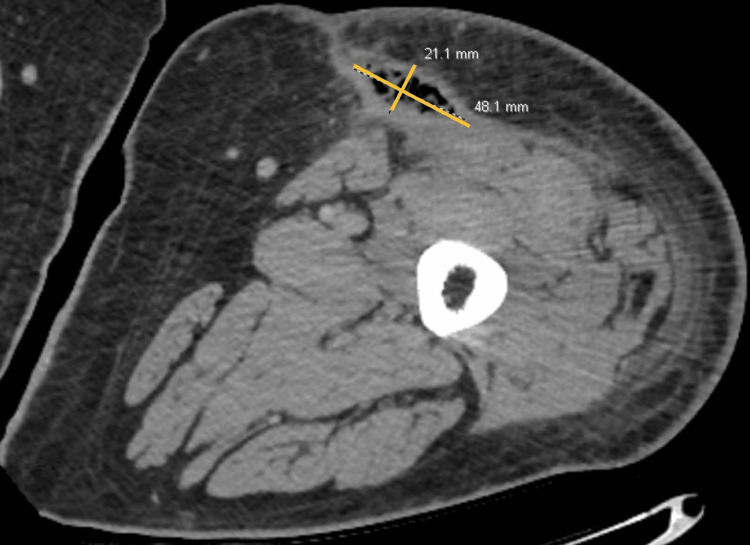
Post-debridement CT left lower extremity shows 2 x 4.8 cm focus of gas contained within the anteromedial thigh.

A week later, he presented with fluid re-accumulation, warmth to the area, fever at home, white count of 12.7 and lactic acid increase from 2.3 to 2.6, random blood sugar 298, and he was readmitted to the hospital for septicemia and drainage with possible wound vac placement. He had I&D of the left leg 50 cm long and 2 cm deep, from the knee to thigh, with similar findings of hematoma collection and was proceeded by wound vac placement (Figures 2, 3). Upon exploring the large wound cavity, poor adhesion of the subcutaneous tissue to the previously sheared muscle fascia was noted. Vicryl sutures in the horizontal mattress fashion were used to reapproximate the patient’s musculature. The cavity was irrigated, and hemostasis achieved with Bovie electrocautery. Two pieces of Adaptic gauze were then placed over the exposed musculature, and a negative pressure dressing applied using a piece of black granulofoam with good seal. He was discharged 10 days later with home health care (HHC), and conservative as well as narcotic pain medications.

He returned a week later with fever, nausea, vomiting, diarrhea, and abnormal drainage from his leg incision. Dehydration was evident on labs, he had acid/base derangements, and his comorbid conditions remained poorly controlled. Surgical incision was done, another wound vac, and partial complex closure, performed five times in the same hospitalization lasting a month complicated by cellulitis/wound infection related to methicillin-resistant Staphylococcus aureus infection. The infectious disease consultant recommended double-strength trimethoprim-sulfamethoxazole 160-800 mg oral tablet for 14 days of therapy at discharge. He remained hospitalized for the full month of December 2022, nearly three months after the initial injury and was discharged as stable again with HHC services. However, he has not been seen since in follow-up or records as of chart review October 2023.

## Discussion

The MLL is an important trauma-related internal degloving injury that is important to follow from a surgical perspective due to its complexity when symptoms persist or worsen that may require surgical intervention. MLLs are often challenging to diagnose, as they can mimic other soft tissue injuries. The key to accurate diagnosis lies in a high index of suspicion, especially in the presence of a history of trauma in the case of our patient. Physical examination findings may include fluctuant, non-reducible swelling with occasional presence of overlying ecchymosis. However, these findings are non-specific and can be easily mistaken for other soft tissue injuries. Imaging modalities play a crucial role in confirming the diagnosis and assessing the extent of the lesion [8]. Ultrasound is often the initial imaging modality of choice as it is readily available and non-invasive and can demonstrate the presence of fluid collections as demonstrated in our patient [8].

Early recognition and prompt management of MLLs are essential to prevent complications such as infection, necrosis, and chronic seroma formation [9]. Treatment options include conservative measures, such as aspiration and compression dressings, as well as more invasive interventions like percutaneous drainage or surgical debridement [7,9]. Conservative management with serial aspirations and compression dressings may be considered for small and acute lesions [9]. However, larger or chronic lesions often require more aggressive interventions as seen in this patient, who required serial wound vacs. Percutaneous drainage can be performed under imaging guidance, and it is effective in evacuating the seroma while minimizing the risk of infection [7]. In cases where conservative or percutaneous drainage fails, surgical exploration with debridement and primary closure may be necessary [7].

Despite the timely identification of the MLL through the utilization of ultrasound and CT imaging in our patient's case, the subsequent management proved to be challenging due to the persistent and complex nature of this particular MLL. Despite initiating multiple incision and drainage procedures followed by VAC placement, our patient experienced a complicated hospital course with ongoing challenges. Despite early intervention, the patient's condition ultimately necessitated multiple surgical debridements, washouts, and serial closure procedures due to chronic recurring seroma formation.

Although MLLs are uncommon, it is imperative for physicians to maintain awareness of this disease entity. This case report's main objective is to make a meaningful contribution to the literature on MLLs, specifically by highlighting the importance of early intervention in an attempt to mitigate complications and improve patient outcomes. Additionally, we seek to provide insights into the complexities involved in managing MLLs despite intensive treatment, further emphasizing the critical role of timely intervention in optimizing clinical outcomes for affected patients.

## Conclusions

The patient in our case presented with clinical manifestations suggestive of an MLL, which was subsequently confirmed through ultrasound and CT imaging. Despite prompt intervention and recognition, the patient's hospital course was remarkably complicated, necessitating intricate surgical and procedural interventions. MLLs can pose a diagnostic challenge due to their nonspecific presentation, and their management can subsequently prove to be even more complex in nature. When left undiagnosed or untreated, these lesions are associated with considerable morbidity. Therefore, we emphasize the importance for clinicians to remain vigilant and knowledgeable about MLLs for early detection and timely management. 

## Appendices

**Table 1:** Types and description of Morel-Lavallee lesions. Original table is created by the authors from information provided in the study by Bonilla-Yoon et al. [1].

Type	Description
1	Serohematic effusion, seroma
2	Subacute hematoma
3	Chronic organizing hematoma
4	Perifascial dissection with closed fatty laceration
5	Perifascial pseudonodular lesion
6	Infected lesion with sinus tract formation, internal septations, thick enhancing capsule
